# An Update on Pain Management in Pediatric Spine Surgery

**DOI:** 10.1007/s12178-026-10037-8

**Published:** 2026-06-01

**Authors:** Sara N. Kiani, Katherine Bach, Vivian Kwok, Ishaan Swarup

**Affiliations:** 1 Orthopaedic Surgery, University of California San Francisco, San Francisco, CA 94143 USA; 2 School of Medicine, University of California San Francisco, San Francisco, CA 94143 USA; 3 Pediatric Orthopaedic Surgery, UCSF Benioff Children’s Hospital, Oakland, CA 94609 USA; 4 UCSF Benioff Children’s Hospital, 747 52nd Street, Oakland, CA 94609 USA

**Keywords:** Pain, Spine surgery, Regional blocks, Anesthesia, Postoperative pain, Pediatric

## Abstract

**Purpose of Review:**

This paper aims to provide an overview on current recommendations for perioperative pain control for pediatric spine surgery. Managing pediatric pain is important to decrease postoperative morbidity, improve early mobilization, and decrease narcotic analgesic use. We aimed to summarize the data on preoperative pain management, intraoperative pain management, and postoperative pain management pathways for pediatric spine surgery.

**Recent Findings:**

Pre-operative mental health conditions have been shown to be associated with increased post-operative pain. Intravenous ketorolac and oral gabapentin use postoperatively can improve postoperative pain control and decrease opioid use. While patient-controlled anesthesia is currently widespread after pediatric spine surgery, there is increasing research on regional modalities for pain control, including erector spinae plane blocks, liposomal bupivacaine, epidural analgesia, and intrathecal morphine injections, which are associated with earlier mobilization and decreased narcotic use, with maintained or decreased pain scores.

**Summary:**

Multimodal pain control, as outlined in Enhanced Recovery After Surgery protocols, is necessary to achieve adequate pain control while decreasing narcotic usage and the associated side effects. Psychosocial factors can impact pain through anxiety and pain catastrophizing. There is increased emphasis on regional and neuraxial anesthesia modalities for pain control. Further research is needed directly comparing the effectiveness of these modalities and further exploring the effect of psychosocial factors on pain and methods to address this.

## Introduction

The incidence of surgical correction for spinal deformities among pediatric patients, most commonly for idiopathic scoliosis, has nearly doubled within the first two decades of the 21st century [1, 2]. Posterior spinal fusion (PSF) and revision of spinal fusion are among the most common pediatric orthopedic procedures overall, trailing only behind fixation for supracondylar humerus fractures [3, 4]. PSF for neuromuscular scoliosis, the second most common spinal deformity requiring surgery, in particular is also among the top procedures with increased rates of adverse outcomes and complications, including persistent pain [2–4]. Enhanced Recovery After Surgery (ERAS) protocols, which incorporate multimodal analgesia in the perioperative periods, have been shown to significantly reduce postoperative pain and may even promote cost savings [2, 5].

While pain management for spine surgery in the adult population has been well reviewed [6], pain management in the pediatric population presents a unique challenge. In addition to differences in drug metabolism, the difficult assessment of pain in children can further impair subsequent planning and management [7]. Moreover, a large subset of pediatric patients with neuromuscular conditions requiring spine surgery presents with cognitive or global developmental delay, making expression of pain challenging [8].

Current pre-operative pain management for PSF emphasizes multimodal analgesia that targets multiple pain pathways, reduces perioperative use of opioids, and improves postoperative pain control [9, 10]. Pre-operative pain management typically incorporates acetaminophen, NSAIDs, gabapentin, and cautious use of opioids. Peri-operative and post-operative pain management further incorporates regional and neuraxial techniques and patient-controlled analgesia. This review paper broadly captures the current landscape of preoperative and peri-operative pain management for spine surgery for pediatric patients.

## Preoperative Pain Management

Nearly 40% of children with idiopathic scoliosis report low back pain [11]. In both Adolescent Idiopathic Scoliosis (AIS) and neuromuscular scoliosis, it is important to first clarify the source of the back pain. Back pain in scoliosis is often multifactorial, with contributions from mechanical stress on discs, facets, and ligaments caused by the curvature, muscular imbalance, postural deconditioning, and psychological factors. Myofascial pain, hip or pelvic pain, and pain from brace wear are other frequent culprits. It is not uncommon for the magnitude of pain to be disproportionate to the curve magnitude; the pain severity does not reliably track with the curve size [12]. Radicular symptoms are uncommon in scoliosis, and it is important to investigate possible additional pathology that could lead to peripheral nerve compression.

The mainstays of treatment for chronic back pain prior to spinal fusion are physical therapy, conditioning, medications – including acetaminophen, non-steroidal anti-inflammatory medications (NSAIDs), and gabapentin – and psychological preparation for future surgery. Physical therapy and conditioning focuses on core strength, spine stabilization, and hamstring stretching. There is some research to suggest that specific exercise programs, like the physical therapeutic scoliosis-specific exercise (PSSE), can reduce pain, increase flexibility, and improve function, spine curvature, and posture [13–15]. Perhaps just as importantly, pre-operative activity modification and physical therapy can help children feel more prepared for the upcoming demands of post-operative recovery.

### Tylenol

Pharmacologic pain management in pediatric patients is broadly categorized between traditional non-opioid analgesics, such as ibuprofen and acetaminophen, and opioids [7]. Common medications used for pain in the pediatric population in the perioperative setting include acetaminophen, NSAIDs, gabapentin, and opioids. The World Health Organization (WHO) pain ladder prioritizes use of non-opioid analgesics, such as acetaminophen and NSAIDs [7]. Acetaminophen is a mainstay of pharmacologic treatment for back pain, and it is a safe first-line medication for children. Dosing is typically 10-15 mg/kg every 4–6 h, but no more than 5 doses in 24 h.

### Nonsteroidal Anti-Inflammatory Drugs

The most common NSAIDs used for children with back pain are ibuprofen, naproxen, and celecoxib. These medications are frequently used in conjunction with acetaminophen. There is some variability in clinical practice regarding discontinuation of NSAIDs prior to surgery due to concerns with bleeding and the efficacy of fusion. Evidence has shown that short-term (< 2 weeks) NSAID usage perioperatively does not increase nonunion rates in spinal fusion surgery [16, 17]. Even so, many clinicians avoid high dose or long-term NSAIDs pre-operatively out of caution. If incorporated in the preoperative plan, NSAIDS should be stopped at least seven days prior to surgery, given concerns with increased bleeding risk.

### Gabapentin

Gabapentinoids were originally approved for use in the prevention of seizures but have widespread use in clinical practice for pain management in children and adults and are particularly useful for neuropathic pain. Gabapentin is often reserved for pediatric patients with more significant pre-operative pain, if a combination of acetaminophen and NSAIDs have been ineffective for pain management. It is more frequently used in neuromuscular scoliosis populations. There are multiple randomized controlled trials that have demonstrated a reduction in opioid consumption and post-operative pain scores when gabapentin is given pre- and post-operatively as part of multimodal regimens [18]. The literature has not demonstrated significant differences in adverse effects for patients receiving gabapentinoids versus control groups. As will be addressed below, ‘gabapentin loading’ is a component of many pre-operative ERAS protocols.

### Muscle Relaxants

Children with neuromuscular scoliosis are also often already on a regimen of muscle relaxant and spasticity medications including baclofen and benzodiazepines, as well as anti-epileptic medications for concurrent seizure syndromes. Muscle relaxants are less commonly needed in patients with AIS.

### Opioids

If possible, pre-operative use of opioid medications for back pain should be avoided. There is extensive literature showing that pre-operative opioid use is associated with increased post-operative opioid needs, longer length of stay, and higher risk for chronic opioid use [19, 20]. Pre-operative opioid use is the most significant predictor of prolonged post-operative opioid use [21]. Pediatric chronic pain management literature similarly demonstrates that opioids have limited evidence for long-term use in children with chronic non-cancer pain, and carry risks of tolerance, dependence, and prolonged use. If patients are already taking opioid medications, it is important to partner with the anesthesia and pain teams leading up to spine surgery to create an individualized plan for optimizing post-operative pain control, which may involve tapering down preoperative opioid use.

### Pre-Operative Medication Pathways

More recently, literature has focused on opioid-sparing pre-emptive pain medication protocols prior to PSF surgery. By initiating pain medication multiple days prior to surgical incision, effective steady state plasma concentrations of each medication can be achieved before surgery. Poon et al. utilized weight-based acetaminophen, celecoxib, and gabapentin starting 48 h prior to PSF for scoliosis and found that these patients had significantly lower post-operative pain scores and a shorter length of inpatient stay after surgery [18]. Many ERAS protocols now incorporate pre-emptive multimodal pain medications into the pain control pathway.

### Psychological Evaluation

One of the most important, and often least addressed, aspects of recovery after pediatric spine surgery is a child’s readiness for surgery from a psychological and mental health perspective. It is well documented that anxiety, depression, a tendency towards pain catastrophizing, and parental anxiety are associated with higher intensity pain post-operatively and worse outcomes after surgery [22–26]. Screening for mental health conditions like anxiety and depression can be helpful in identifying children who would likely benefit from dedicated psychiatric or psychological support perioperatively. There are also screening tools to assess a child’s self-efficacy and likelihood of pain catastrophizing.

Regardless of pre-operative mental health, undergoing spine surgery is extremely challenging and can lead to various psychological struggles [27–29]. Research has shown that children who are mentally prepared for surgery and have more support throughout their hospitalization recover more quickly and have fewer emotional problems like separation anxiety and sleep disturbances, than those who were less prepared [30]. Focusing on thorough pre-operative education to prepare children and families for the inpatient experience is crucial to allay anxiety [31, 32]. It can be helpful to include videos of a “hospital tour” showing the pre-operative area, operating room, and inpatient floor, so that children know what to expect on the day of their surgery. Additionally, engaging children with psychological interventions perioperatively, like mindfulness, meditation, relaxation/breathing techniques, coping skills, and cognitive behavioral therapy has been shown to reduce persistent post-operative pain and physical impairment [33, 34]. Incorporating education about coping skills and mindfulness pre-operatively can lower post-operative pain and anxiety in adolescents undergoing spinal fusion [31, 35].

## Perioperative Pain Management

Many of the medications used in the pre-operative setting described above can also be utilized during the peri-operative setting. Managing postoperative pain is important to decrease postoperative morbidity, improve early mobilization, and decrease narcotic analgesic use [36].

In addition to their established role in the preoperative setting, acetaminophen and NSAIDs remain foundational components of multimodal analgesia throughout the perioperative period. Acetaminophen serves as a foundational peri-operative non-opioid analgesic with a favorable safety profile [7, 37]. Perioperative administration, particularly intravenous acetaminophen, offers reliable bioavailability and has also been associated with improved pain control [38]. However, pediatric spine-specific data suggest a more nuanced benefit regarding its impact on post-operative opioid consumption [38].

When used in combination with opioids, the addition of NSAIDs results in improved pain management while also decreasing consumption of post-operative opioids [7, 37–39]. Despite concern for impaired bone healing, current pediatric literature does not demonstrate an increased risk of pseudarthrosis following PSF for AIS [17, 38]. A review including biochemical, animal, human studies found that appeared to be an effect, but this is dose- and duration-dependent and the authors recommend limiting NSAID use to short perioperative usage [16]. In practice many surgeons order a short course of intravenous ketorolac perioperatively to balance pain control with the theoretical risk of pseudoarthrosis. Similarly, perioperative administration of NSAIDs has also not been associated with clinically significant increases in bleeding or transfusion requirements among pediatric patients following surgery for scoliosis [38].

Another non-opioid medication commonly used in the perioperative setting is gabapentin. Its inclusion as part of a multimodal regimen has been associated with improved postoperative pain control after PSF. In some studies, gabapentin when used as part of a multimodal pain regimen has also been shown to significantly decrease opioid consumption following PSF among patients with AIS [18, 40–42]. However, its effect on opioid requirements may be limited or variable, depending on dosing and the timepoint in the post-operative period [38].

Benzodiazepine usage has not been shown to reduce postoperative pain scores or decrease postoperative opioid use in adults [43]. One study of over 7,000 patients undergoing PSF for AIS found that benzodiazepine use was associated with greater odds of prolonged IV opioid use and length of stay [44]. This data should be considered cautiously, as it may be strongly mediated by psychosocial factors that may increase opioid use and length of stay regardless. Given the side effect profile of these medications with limited evidence supporting their use, they should be prescribed thoughtfully. These may be useful for patients with neuromuscular scoliosis, where there has not been significant research, or after surgeries requiring large corrections.

Furthermore, while more controversial given adverse side effect profile, use of opioid medications has also been shown to be effective among pediatric patients undergoing spine surgery. When used intraoperatively as part of ERAS pathways, methadone has been shown to achieve pain management without increasing use of post-operative opioids [45, 46]. Scheduled methadone may maintain pain scores and reduce total opioid consumption without increasing sedation after pediatric PSF [47, 48]. A review of the data on perioperative methadone use in children and adolescents suggests the development of procedure-specific methadone protocols and additional studies on the safety and efficacy as the primary opioid for pain management postoperatively [46].

### Patient Controlled Anesthesia

Opioids are often initially used in the form of intravenous morphine or hydromorphone Patient Controlled Anesthesia (PCA), with one multicenter study finding that 99.9% of patients received a PCA postoperatively [36]. In addition to the long-term risk of chronic opioid use, there are acute adverse events that must be monitored, including respiratory depression, pruritus, and nausea and vomiting. Usage is typically highest in the first 12 h before declining progressively, which follows the postoperative pain pattern which peaks at 12 h postoperatively [49]. This suggests that as part of efforts to decrease narcotic use, PCA use should be limited and discontinued on postoperative day one. Data supports use of “bolus only” administration with lower morphine consumption and similar pain scores as “basal and bolus” settings [50].

### Erector Spinae Plane Blocks/Liposomal Bupivacaine

Given early postoperative pain is often a significant problem, methods to decrease early narcotic use while maintaining adequate pain control have been discussed. The ultrasound-guided erector spinae plane block (ESPB) was initially developed for managing thoracic neuropathic pain and has been shown to be effective in many surgeries, including pediatric spine surgery [51]. A prospective double-blinded randomized controlled trial found lower numerical rating scale pain scores at all time points within 48 h of surgery, decreased total opioid consumption, and lower surgery-induced stress response as measured by neutrophil-to-lymphocyte and platelet-to-lymphocyte ratios [52]. There was no effect on intraoperative motor evoked potentials [52, 53].

Retrospective reviews have found similar efficacy. In a retrospective case-control study, Akesen et al. also showed reduced usage of rescue analgesics and higher patient satisfaction [54]. Other studies have shown decreased postoperative opioid use, fewer days to first ambulation and decreased laxative use, without an increase in postsurgical pain or block related complications [55–57].

One cited shortcoming is the duration of action may not be sufficient particularly given the length of some pediatric spine surgeries. ESPB catheters have been described but these could not be performed preoperatively and, like single shot postoperative ESPB, would need to be performed prior to dressing placement which theoretically may impact sterility of the incision and would disrupt typical postoperative workflow [58].

One alternative solution is intrafascial dexamethasone as an adjuvant to ESPB, which has been shown to prolong analgesia after pediatric spine surgery and reduce opioid requirements in the first 48 h postoperatively with no increase in adverse effects, including no differences in perioperative blood glucose levels or neurological complications [59].

Liposomal bupivacaine was recently approved for infiltration and fascial plane blocks in pediatric patients aged 6 years or older. The liposomes slowly metabolize releasing bupivacaine over multiple days. Surgical site administration of liposomal bupivacaine has been shown to decrease opioid use – both overall and specifically intravenous opioid usage –, with similar or decreased pain scores, similar or increased postoperative mobility, and similar side effects [60–62].

### Epidural Analgesia/Intrathecal Morphine

Epidural analgesia is another pain management option that has also been shown to reduce systemic narcotic use. A multicenter study of over 2,000 patients found epidural and spinal opioid groups had lower total opioid requirements compared with PCA, without significant differences in pain scores, complications, or length of stay [63]. In a comparison with PCA, continuous epidural analgesia (CEA) showed earlier postoperative defecation and mobilization and shorter length of stay [64]. There was transient neurological irritation in 9.7% of patients in the epidural group, but this resolved spontaneously in all patients. One randomized controlled trial showed that patient-controlled intermittent epidural bolus can be used instead of continuous infusion with lower morphine consumption and fewer side effects [65].

Intrathecal morphine (ITM) has also been shown to provide effective pain control after AIS surgery with decreased opioid use, later first opioid use postoperatively, earlier mobilization, and decreased length of stay when compared to standard postoperative pathways (PCAs and non-scheduled intravenous and oral opioids) and epidural infusion [66–71]. ITM has been shown to have benefits when compared to epidural hydromorphone as well, including lower pain scores immediately postop, earlier ambulation, earlier foley removal, and shorter hospital stays [68]. There is also data that shows lower estimated blood loss in patients who have received ITM prior to pediatric spine surgery [72, 73].

Higher doses have been shown to be associated with respiratory depression in some studies but do not lead to measurable improvements in pain control [73, 74]. ICU admission is not typically required with the use of ITM, which both decreases costs and can decrease length of stay. A single-center observational study over 25 years found a small percentage of patients may require ICU admission for respiratory depression, but re-intubation was not required for any patients [70].

There is a risk for cerebrospinal fluid leakage with intrathecal medications. While there is limited data in this population, there have been studies showing a 20–25% risk of CSF leakage with intrathecal baclofen and opioid administration [75, 76]. A review of all cause incidental dural tears from over 3,000 pediatric PSF found that the primary symptom was headaches in 13% of patients and this resolved on average in 1 week [77]. The majority were identified and repaired intraoperatively and there were no noted long term sequelae [77]. The timing of ITM administration (before incision, during exposure, or after deformity correction) has not been shown to significantly impact perioperative pain control, functional recovery or early outcomes, therefore early administration is reasonable which would provide adequate time to identify and repair postoperative dural tears if they occur.

### Postoperative Pain Management Protocols

Postoperative pain control after pediatric spine surgery should be sufficient to allow for ambulation by POD1, hospital stay *≤* 3 days, and minimal use of intravenous opioids. Optimal pain management methods utilize a combination of nonopioid analgesics and regional and neuraxial techniques to minimize opioid use and optimize pain control and function. Current methods focus on the use of spine protocols, most commonly ERAS Pathways and Rapid Recovery Pathways. These have been shown to reduce hospital length of stay by 1–2 days, accelerate functional recovery and decrease opioid consumption without increasing rates of postoperative complications or readmission [78–81].

While there is heterogeneity in the specific recommendations, these pathways often include a combination of the following preoperative, intraoperative, and postoperative recommendations. Preoperatively, appropriate expectation counseling, preoperative gabapentinoid loading, and minimizing fasting time is recommended. Intraoperatively, these pathways emphasize neuraxial opioids, long-acting opioids, local anesthetic infiltration, avoidance of background morphine infusions, use of tranexamic acid, and prophylactic antiemetics and steroids. Postoperatively, patients can benefit from early mobilization, transition to oral meds, early removal of urinary catheters, discontinuation of surgical drains, early physical therapy with ambulation by postoperative day 1, rapid transition to oral analgesics and avoidance or decreased PCA usage, early resumption of oral diet, scheduled non-opioid analgesics, and elimination of routine ICU admission.

New implementation of these pathways is difficult and requires multidisciplinary collaboration between anesthesia, operating room staff, surgeons, nurses, physical therapists, and pharmacy. Standardized order sets can streamline postoperative care and improve implementation. Clear discharge criteria are helpful and can help guide care postoperatively.

### Chronic Postsurgical Pain

Chronic Post-Surgical Pain (CPSP) is defined as pain scores greater than 3 out of 10 on the numerical rating scale beyond two months postop. Meta-analysis shows a 31% post-spine fusion rate of CPSP [82]. CPSP is associated with reduced function and quality of life [36]. Adequate control of perioperative pain may be an important factor in reducing rates of CPSP given that higher preoperative back pain, postoperative pain intensity, and postoperative pain use are all risk factors for CPSP [83, 84]. Psychosocial factors have been shown to contribute to the development of CPSP, with pain catastrophizing mediating part of the effect between acute pain and CPSP [84], again emphasizing the importance of psychological intervention as an important component of preoperative and perioperative pain management.

Looking specifically at pediatric spine surgery, perioperative pregabalin and intraoperative opioid use (versus an opioid sparing approach) have not been shown to change rates of CPSP [83, 85]. A systematic review of multiple surgeries, including spine surgery, showed IV lidocaine, ketamine, and gabapentinoid use reduced the incidence of CPSP and determined that these interventions may be more effective in those with a higher baseline risk.

## Conclusions

Multimodal pain control, as outlined by ERAS pathways, has been shown to provide appropriate postoperative pain control. There is increasing support for neuraxial and regional anesthesia as an adjunct, which has been shown to decrease opioid requirements, increase early mobilization, and decrease length of stay after pediatric spine surgery. Additionally, psychosocial factors, specifically anxiety and pain catastrophizing, have a significant impact on pain after pediatric spine surgery and may be a target for future interventions. There is early data in support of methadone for pain control, but further research on this topic is needed. There is limited data supporting the use of benzodiazepines, but there may be populations in which these medications should be used, and more nuanced data is needed to provide recommendations. Finally, much of the available data focuses on pain control after surgery for AIS, with limited information in the neuromuscular scoliosis population, for which ideal pain control may differ given increased presence of muscle spasms and in some cases an inability to verbally communicate a need for pain medication. There have been many advances in pain control after pediatric spine surgery and increased emphasis on regional and neuraxial methods that may maintain or improve pain scores, while increasing early mobilization and decreasing narcotic use.

## Key References

- Halpern LM, Zhang D-A, Kogan CJ, Ryan KA, Correll Z, Bronson W (2026) A Comparison of Continuous Epidural, Spinal Opioid, and Patient-Controlled Analgesia for Posterior Spinal Fusion for Adolescent Idiopathic Scoliosis: A Multicenter, International Database Study. J Pediatr Orthop. 10.1097/BPO.0000000000003272.
  - This multicenter study of over 2,000 patients found epidural and spinal opioid groups had lower total opioid requirements compared with PCA, without significant differences in pain scores, complications, or length of stay, supporting the trend towards regional and neuraxial anesthesia for pediatric spine surgery.
- Domagalska M, Ciftsi B, Janusz P, Reysner T, Daroszewski P, Kowalski G, Wieczorowska-Tobis K, Kotwicki T (2024) Effectiveness of the Bilateral and Bilevel Erector Spinae Plane Block (ESPB) in Pediatric Idiopathic Scoliosis Surgery: A Randomized, Double-Blinded, Controlled Trial. Journal of Pediatric Orthopaedics 44:e634. 10.1097/BPO.0000000000002707.
  - This reference is the first RCT showing the efficacy of ESPB in pediatric spine surgery, which is growing in popularity and been effective in reducing postoperative opioid use.
- Li R, Rogers AH, Sujan AC, Zhou C, Thiagarajan P, Palermo TM, Kain ZN, Rabbitts JA (2025) Psychosocial mechanisms underlying the transition from acute to chronic postsurgical pain in youth following spinal fusion: a 6-month longitudinal study. Pain 166:e447–e459. 10.1097/j.pain.0000000000003631.
  - This reference shows the impact of psychosocial factors, particularly anxiety and pain catastrophizing, that contribute to the development of chronic pain after spinal fusion.

## Data Availability

No datasets were generated or analysed during the current study.
